# An Empirical Bayes Optimal Discovery Procedure Based on Semiparametric Hierarchical Mixture Models

**DOI:** 10.1155/2013/568480

**Published:** 2013-04-10

**Authors:** Hisashi Noma, Shigeyuki Matsui

**Affiliations:** Department of Data Science, The Institute of Statistical Mathematics, 10-3 Midori-cho, Tachikawa, Tokyo 190-8562, Japan

## Abstract

Multiple testing has been widely adopted for genome-wide studies such as microarray experiments. For effective gene selection in these genome-wide studies, the optimal discovery procedure (ODP), which maximizes the number of expected true positives for each fixed number of expected false positives, was developed as a multiple testing extension of the most powerful test for a single hypothesis by Storey (*Journal of the Royal Statistical Society, Series B,* vol. 69, no. 3, pp. 347–368, 2007). In this paper, we develop an empirical Bayes method for implementing the ODP based on a semiparametric hierarchical mixture model using the “smoothing-by-roughening" approach. Under the semiparametric hierarchical mixture model, (i) the prior distribution can be modeled flexibly, (ii) the ODP test statistic and the posterior distribution are analytically tractable, and (iii) computations are easy to implement. In addition, we provide a significance rule based on the false discovery rate (FDR) in the empirical Bayes framework. Applications to two clinical studies are presented.

## 1. Introduction

The comprehensive, gene expression microarrays are a powerful tool for screening the differentially expressed genes among different phenotypes such as clinical subtypes and prognostic classes of disease from a large pool of candidate genes. Gene screening studies have the potential to be useful for elucidating disease biology and aggressiveness, identifying new therapeutic targets, and developing new molecular diagnostics for optimized medicine for individual patients [1–4]. The high dimensionality of microarray data, however, has posed special challenges in extracting a small number of relevant genes from a large quantity of noise variables by gene screening analysis. In addition to the control of false positives [5–7], improvement of the efficiency of gene screening analysis is important.

For efficient screening of differentially expressed genes, Storey [8] developed the optimal discovery procedure (ODP), which can be interpreted as an extension of the most powerful test for a single hypothesis testing [9] to multiple testing. Storey defined an optimality criterion for multiple testing that maximizes the expected number of true positives (ETP) for each fixed level of expected false positives (EFP) [8]. The ODP was developed as a testing procedure that achieves this optimality; it improves the overall performance of multiple tests by “borrowing strength” across tests. However, in applying the ODP, the following two components must be estimated: (a) the true status of each significance test (null or alternative) and (b) the true probability distribution corresponding to each test [8]. To address these estimation problems, Storey et al. [10] constructed the generalized likelihood ratio statistic [11], using maximum likelihood estimates. Cao et al. [12] also proposed a Bayesian approach, and Woo et al. [13] developed a computationally efficient method called “modular ODP.”

More recently, Noma and Matsui [14] developed an empirical Bayes approach for the ODP that can effectively circumvent the estimation problems of (a) and (b). Under a hierarchical, random effects model, they showed that the ODP was derived as a testing rule based on the marginal likelihood ratio statistic. In their numerical evaluations based on simulations, the empirical Bayes method nearly achieved the theoretical bound of ETP (i.e., average power) and performed well, compared with the existing methods under a broad range of conditions [14].

A critical aspect of this empirical Bayes approach is that a fully parametric form in the hierarchical modelling must be specified. Although most of the empirical Bayes methods previously discussed in microarray studies [15–21] assume fully parametric natural conjugate models for the prior distribution, this assumption might be restrictive because of possible substantial diversity of effect size among informative genes caused by complex molecular pathways, whose distribution is generally unknown in microarray studies. In this article, we develop an empirical Bayes method for the ODP based on a semiparametric hierarchical mixture model, in which the prior distribution for effect sizes is not specified parametrically. We estimate the nonparametric component of the prior distribution by applying the “smoothing-by-roughening” approach [22, 23] and provide an effective computational method for the ODP statistic.

This article is organized as follows. In Section 2, we provide a brief overview of the theoretical results regarding the ODP methods by Storey [8] and Noma and Matsui [14]. In Section 3, we describe the semiparametric hierarchical mixture model and how to implement the ODP method. We present applications to prostate cancer and lymphoma clinical studies in Section 4. Finally, we provide a discussion in Section 5.

## 2. The Optimal Discovery Procedure in Multiple Significance Testing

### 2.1. Storey's Optimal Discovery Procedure

In this section, we briefly review the theoretical results regarding the ODP methods. We denote the datasets from *m* genes as **x**_1_, **x**_2_,…, **x**_*m*_, where **x**_*k*_ = (*x*_*k*1_, *x*_*k*2_,…,*x*_*kn*_)^*T*^  (*k* = 1,…, *m*). In microarray studies, **x**_*k*_ corresponds to a set of log-transformed, normalized gene expressions from *n* samples for the *k*th gene (see Section 4). Also, suppose that the datasets are random vectors defined in a common probability space. The optimal goal when identifying differentially expressed genes is to find as many true positives as possible, without incurring too many false positives [10]. Storey [8] defined an optimal criterion of multiple testing as a rule of statistical significance that maximizes ETP for a certain fixed EFP level.

He derived the following lemma that gives the multiple testing procedure that achieves the optimal criterion.

**Lemma 1 (Storey [8])**

Suppose the *k*th test has a null density *f*_*k*_(**x**) and alternative density *g*_*k*_(**x**)  (*k* = 1,…, *m*). Also, assume that the null hypothesis is true for the *k*th test (*k* = 1, …, *m*_0_) and the alternative is true for *k* = *m*_0_ + 1, …, *m*, without loss of generality. The following significance threshold statistic achieves the ODP criterion:

(1) $$\begin{array}{c} S_{\text{ ODP }}\left(x\right)=\frac{g_{m_{0}+1}\left(x\right)+g_{m_{0}+2}\left(x\right)+\cdots +g_{m}\left(x\right)}{f_{1}\left(x\right)+f_{2}\left(x\right)+\cdots +f_{m_{0}}\left(x\right)}. \end{array}$$

Given a fixed cut-off *λ*  (0 ≤ *λ* < *∞*) chosen to attain an acceptable EFP level, the null hypothesis for the *k*th test is rejected if and only if *S*_ ODP _(**x**_*k*_) ≥ *λ*.

It should be noted that the ODP statistic is composed of all the density functions on *m* tests. In other words, through borrowing strengths across *m* tests, the overall power is improved more than the conventional most powerful test for single hypothesis tests [9, 11]. Also, the optimality result is held regardless of correlation structure among the datasets **x**_1_, **x**_2_,…, **x**_*m*_.

As mentioned in Section 1, for applying *S*_ODP_(**x**), there are two practical issues: (a) estimation of the true status of each hypothesis (null or alternative), corresponding to the numerator and denominator of *S*_ODP_(**x**), and (b) estimation of the true probability density functions *f*_*k*_(**x**)'s and *g*_*k*_(**x**)'s at which **x**_*k*_'s are evaluated. Storey et al. [10] provided an practical estimating method, motivated by the generalized likelihood ratio test for single significance tests [11]. They noticed that the significance rule based on

(2) SODP(x)+1  =f1(x)+⋯+fm0(x)+gm0+1(x)+⋯+gm(x)f1(x)+f2(x)+⋯+fm0(x)

is equivalent to that of *S*_ODP_(**x**) in Lemma 1. For each test *k* = 1,…, *m*, let ${\hat{f}}_{k}(x)$ be an estimate of the null density function with all the unknown parameters replaced by their maximum likelihood estimates under the constraints of the null hypothesis, and let ${\hat{g}}_{k}(x)$ be the analogous estimate for the alternative hypothesis using the unconstrained maximum likelihood estimates. Then, Storey et al. [10] proposed to use the canonical plug-in estimate:

(3) $$\begin{array}{c} {\hat{S}}_{\text{ODP}}\left(x\right)=\frac{{\hat{g}}_{1}\left(x\right)+{\hat{g}}_{2}\left(x\right)+\cdots +{\hat{g}}_{m}\left(x\right)}{{\hat{w}}_{1}{\hat{f}}_{1}\left(x\right)+{\hat{w}}_{2}{\hat{f}}_{2}\left(x\right)+\cdots +{\hat{w}}_{m}{\hat{f}}_{m}\left(x\right)}, \end{array}$$

where ${\hat{w}}_{k}\;\;(k=1,2,\ldots ,m)$ is an estimate of the status of the *k*th hypothesis. For estimating ${\hat{w}}_{k}$, Storey et al. [10] ranked the tests based on a univariate statistic (e.g., the *t*-statistic for two-class comparison) and estimated it as ${\hat{w}}_{k}=1$ when the *k*th test fell below the estimated proportion of true nulls by Storey [6], and ${\hat{w}}_{k}=0$ otherwise. Other estimating procedures for *S*_ODP_(**x**) were also proposed by Cao et al. [12] and Woo et al. [13].

### 2.2. Empirical Bayes Approach by Noma and Matsui [14]

As Storey [8] pointed out, the ODP is analogous to the well-known shrinkage estimation for multiple-point estimation [24, 25]. Because the shrinkage estimation is interpreted as the empirical Bayes estimation [26–28], hierarchical modelling would be a natural formulation for information sharing across tests, both in deriving the ODP and in developing estimation methods for implementing it [14].

Suppose that the probability density functions for **x**_1_, **x**_2_,…, **x**_*m*_ have the same parametric form *f*(**x** | ***θ***_*k*_, **ψ**_*k*_), where ***θ***_*k*_ is the parameter of interest and **ψ**_*k*_ is the nuisance parameter for the *k*th test. We assume the following prior distribution for the parameters (***θ***_*k*_, **ψ**_*k*_):

(4) $$\begin{array}{c} \text{null}:\;\;\left(\theta_{\text{k}},\psi_{\text{k}}\right)\sim G_{0}\left(\theta ,\psi \;|\;\xi_{0}\right),\\ \text{alternative}:\;\;\left(\theta_{\text{k}},\psi_{\text{k}}\right)\sim G_{1}\left(\theta ,\psi \;|\;\xi_{1}\right), \end{array}$$

where **ξ**_0_ and **ξ**_1_ are the hyperparameters of the prior distribution.

**Lemma 2 (Noma and Matsui [14])**

Under the empirical Bayes framework with the prior distribution (4), the following significance threshold function achieves the ODP criterion:

(5) R
ODP
(x)=EG1[f(x ∣ θ,ψ)]EG0[f(x ∣ θ,ψ)]=∫f(x ∣ θ,ψ)dG1(θ,ψ ∣ ξ1)∫f(x ∣ θ,ψ)dG0(θ,ψ ∣ ξ0).

For a fixed cut-off *λ*  (0 ≤ *λ* < *∞*) chosen to reach an acceptable EFP level, the null hypothesis for the *k*th test is rejected if and only if *R*_ ODP _(**x**_*k*_) ≥ *λ*.

See Noma and Matsui [14] for interpretations of *R*_ODP_(**x**).

For the specification of the prior distributions *G*_0_(***θ***, **ψ** | **ξ**_0_) and *G*_1_(***θ***, **ψ** | **ξ**_1_), Noma and Matsui [14] adopted the empirical Bayes method [26, 27]. The empirical Bayes implementation is used to obtain estimates for **ξ**_0_ and **ξ**_1_ from the data, which can then be substituted into the ODP statistic, *R*_ODP_(**x**),

(6) $$\begin{array}{c} {\hat{R}}_{\text{ODP}}\left(x\right)=\frac{\int f\left(x\;|\;\theta ,\psi \right)dG_{1}\left(\theta ,\psi \;|\;{\hat{\xi }}_{1}\right)}{\int f\left(x\;|\;\theta ,\psi \right)dG_{0}\left(\theta ,\psi \;|\;{\hat{\xi }}_{0}\right)}, \end{array}$$

where ${\hat{\xi }}_{0}$ and ${\hat{\xi }}_{1}$ are the estimators of **ξ**_0_ and **ξ**_1_, respectively.

In comparison with the existing ODP estimation methods based on *S*_ODP_(**x**), the empirical Bayes approach has two advantages. First, it estimates only the hyperparameters **ξ**_0_ and **ξ**_1_ for the random effects models, and not the parameters in the density function **x**_*k*_  (*k* = 1,…, *m*), for which a large number of parameters (thousands or more in microarray studies) must be estimated as in the procedure of Storey et al. [10]. Second, in the empirical Bayes approach, the estimation of the true status, null or alternative, for each test is not needed.

## 3. Semiparametric Hierarchical Mixture Model and the ODP

### 3.1. Semiparametric Hierarchical Mixture Model

In this section, we consider applying the ODP to concrete microarray analyses of two-class comparisons (with classes denoted as “0” and “1”), for example, good prognosis and poor prognosis, on the basis of the expression levels of *m* genes from *n* samples. The gene expression data considered here comprise normalized log ratios from two-color complementary DNA arrays or normalized log signals from oligonucleotide arrays (e.g., Affymetrix GeneChip). Let *n*_0_ and *n*_1_ be the sample sizes of classes 0 and 1, respectively. Let *μ*_0*k*_ and *μ*_1*k*_  (*k* = 1,2,…, *m*) be the means of the gene expression levels for classes 0 and 1, respectively. We consider the detection of differentially expressed genes to be when *μ*_0*k*_ ≠ *μ*_1*k*_. That is, testing problem for

(7) H0k:μ0k=μ1k  versus    H1k:μ0k≠μ1k(k=1,2,…,m).

We assume that gene expressions of the *k*th gene are normally distributed within each class,

(8) $$\begin{array}{c} x_{k1},\ldots ,x_{kn_{0}}\sim N\left(\mu_{0k},\sigma_{k}^{2}\right),\\ y_{k1},\ldots ,y_{kn_{1}}\sim \text{N}\left(\mu_{1k},\sigma_{k}^{2}\right), \end{array}$$

where *σ*_*k*_^2^ is a common within-class variance (*k* = 1,2,…, *m*), as in Storey et al. [10]. Let *d*_*k*_ be the difference of the mean of the two classes for the *k*th gene (*k* = 1,2,…, *m*), *μ*_1*k*_ − *μ*_0*k*_, which is the parameter of interest. Let ${\overset{-}{x}}_{k}$, ${\overset{-}{y}}_{k}$ denote the sample means of the gene expression levels for classes 0 and 1, respectively. We consider the following translation:

(9) $$\begin{array}{c} w_{ki}=\sqrt{\frac{n_{0}}{n_{0}-1}}\left(x_{ki}-{\overset{-}{x}}_{k}\right),\\ z_{ki}=\sqrt{\frac{n_{0}}{n_{0}+1}}\left(y_{ki}-{\overset{-}{x}}_{k}\right). \end{array}$$

Using this translation, the nuisance parameters *μ*_0*k*_'s are eliminated in the models of *w*_*ki*_ and *z*_*ki*_. Specifically,

(10) $$\begin{array}{c} w_{k1},\ldots ,w_{kn_{0}}\sim N\left(0,\sigma_{k}^{2}\right),\\ z_{k1},\ldots ,z_{kn_{1}}\sim N\left(\theta_{k},\sigma_{k}^{2}\right), \end{array}$$

where $\theta_{k}=\sqrt{n_{0}/\left(n_{0}+1\right)}d_{k}$. Note that the testing problems in (7) are equivalent to testing

(11) H0k':θk=0  versus  H1k':θk≠0(k  =  1,2,…,m).

Let **w**_*k*_ = (*w*_*k*1_,…, *w*_*kn*_0__) and **z**_*k*_ = (*z*_*k*1_,…, *z*_*kn*_1__).

For applying the ODP to the testing problems *H*_0*k*_′ : *θ*_*k*_ = 0  (*k* = 1,2,…, *m*) under model (10), we assume the following random effects distributions for the model parameters (*θ*_*k*_, *σ*_*k*_^2^):

(12) $$\begin{array}{c} \theta_{k}\sim \pi_{0}\delta \left(\theta \right)+\pi_{1}G\left(\theta \right),\\ \sigma_{k}^{2}\sim {\text{Gamma}}^{-1}\left(\alpha ,\beta \right),\\ \left[p\left(\sigma_{k}^{2}\right)=\frac{\beta^{\alpha }}{\Gamma \left(\alpha \right)}\sigma_{k}^{-2(\alpha +1)}\exp\left(-\frac{\beta }{\sigma_{k}^{2}}\right)\right], \end{array}$$

where *δ*(*θ*) in the first component of *θ*_*k*_ is the Dirac delta function, representing nondifferential expression between two classes. The second component of *θ*_*k*_ represents a component of the differential expression, and *π*_0_ and *π*_1_ are the mixing proportions for the components (*π*_0_ + *π*_1_ = 1). The random effects distribution of the gene-specific variances *σ*_*k*_^2^ is specified as the parametric conjugate model for analytical tractability. For modeling *G*(*θ*), instead of adopting a fully parametric conjugate normal distribution model as done by Noma and Matsui [14], we apply the “smoothing-by-roughening” approach of Laird and Louis [22] and Shen and Louis [23]. This approach was proposed to obtain the nonparametric maximum likelihood estimate of the prior distribution in empirical Bayes analysis [29, 30]. This approach starts with a smooth estimate of the prior distribution and uses the EM algorithm to “roughen” it towards the nonparametric maximum likelihood estimate. For modeling *G*(*θ*), we place supports at equally spaced discrete mass points at a series of nonzero points **a** = (*a*_1_, *a*_2_, …, *a*_*L*_), for sufficiently large values of *L*. We assume that

(13) $$\begin{array}{c} \text{Pr}\left(\theta =a_{j}\right)=p_{j}\;\left(j=1,2,\ldots ,L\right), \end{array}$$

where *p*_1_ + *p*_2_ + ⋯+*p*_*L*_ = 1. We denote the hyperparameters of the distribution function for the differential component as **p** = (*p*_1_, *p*_2_,…, *p*_*L*_). Shen and Louis [23] provided a detailed guideline for implementing the smoothing-by-roughening approach based on their numerical evaluations. Shen and Louis [23] recommended that the number of grid points should be at least 200. For simplicity, they suggested the grid points can be equally spaced on the support of the distribution. Increasing the number of grid points and spacing them according to the prior density will provide a better approximation of *G*(*θ*) and therefore will improve the estimation, particularly for the tail areas. However, too large numbers might not necessarily be beneficial. For more details involving analytical and numerical evaluations, see the guideline in Shen and Louis [23]. In this article, we refer to this hierarchical model as the “semiparametric hierarchical mixture model.” The important property of this model is that the posterior distributions of *θ*_*k*_ and *σ*_*k*_^2^  (*k* = 1,2,…, *m*) can be obtained analytically. We provide the analytical representation of the posterior distributions in Section 3.4.

The maximum likelihood estimates of the hyperparameters **η** = (*π*_0_, *π*_1_, **p**, *α*, *β*) are obtained by the EM algorithm [31]. Because the posterior distributions of *θ*_*k*_ and *σ*_*k*_^2^ are obtained analytically, the E-step of the EM algorithm can be implemented easily. The details of the EM algorithm are presented in the appendix.

### 3.2. The Optimal Discovery Procedure

The ODP statistic *R*_ODP_(**w**, **z**) for the testing problems (11) is obtained as the marginal likelihood ratio. The marginal likelihood of the null hypothesis corresponds to the marginal likelihood of the nondifferential component:

(14) h0(wk,zk)  =∫0∞p(wk,zk ∣ θk=0,σk2)p(σk2 ∣ α,β)dσk2  =Γ(n/2+α)Γ(α)βα(2π)−n/2   ×(∑i=1n0wki2+∑i=1n1zki22+β)−(n/2+α).

Similarly, the marginal likelihood of the alternative hypothesis in (11) corresponds to that of the differential component:

(15) h1(wk,zk)  =∑j=1L∫0∞p(wk,zk ∣ θk=aj,σk2)       ×p(σk2 ∣ α,β)dσk2Pr(θk=aj)  =Γ(n/2+α)Γ(α)βα(2π)−n/2   ×∑j=1L(∑i=1n0wki2+∑i=1n1(zki−aj)22+β)−(n/2+α)pj

under the semiparametric hierarchical mixture model.

Thus, the ODP statistic (the marginal likelihood ratio) is given as

(16) RODP(wk,zk)  =(∑j=1L∫0∞p(wk,zk ∣ θk=aj,σk2)        ×p(σk2 ∣ α,β)dσk2Pr(θk=aj))   ×(∫0∞p(wk,zk ∣ θk=0,σk2)p(σk2 ∣ α,β)dσk2)−1  =(∑j=1L(∑i=1n0wki2+∑i=1n1(zki−aj)22+β)−(n/2+α)pj)   ×((∑i=1n0wki2+∑i=1n1zki22+β)−(n/2+α))−1.

By plugging-in the maximum likelihood estimate of **η**, the empirical Bayes testing procedure is implemented based on

(17) R^ODP(wk,zk)=∑j=1L((∑i=1n0wki2+∑i=1n1(zki−aj)2)/2+β^)−(n/2+α^)p^j((∑i=1n0wki2+∑i=1n1zki2)/2+β^)−(n/2+α^).

Note that the ODP statistic is obtained in closed form using the maximum likelihood estimate of **η**.

### 3.3. Assessing Significance

For assessing significance, we can obtain an empirical Bayes estimator of FDR [5–7] under the semiparametric hierarchical mixture model as follows. For any given threshold *λ*, we denote the significant region Λ(*λ*) = {(**w**, **z**) | *R*_ODP_(**w**, **z**) ≥ *λ*} for each test. Let *Ξ* denote the set of indices for the significant genes. In the framework of Bayesian selection rules, the FDR accords to the misclassification error rate of differential/non-differential classification [6, 7, 32], and one of the well-known Bayesian estimator of the FDR [33, 34] is

(18) $$\begin{array}{c} \hat{\text{FDR}}\left(\lambda \right)=\frac{1}{\left|\Xi \right|}\sum_{k\in \Xi }\text{Pr}\left(H_{0k}\;|\;w_{k},z_{k}\right), \end{array}$$

where

(19) Pr(H0k ∣ wk,zk)=π0h0(wk,zk)π0h0(wk,zk)+π1h1(wk,zk)=(π0(∑i=1n0wki2+∑i=1n1zki22+β)−(n/2+α)) ×(π0(∑i=1n0wki2+∑i=1n1zki22+β)−(n/2+α)   +π1∑j=1L(∑i=1n0wki2+∑i=1n1(zki−aj)22+β)−(n/2+α)pj)−1.

Accordingly, an empirical Bayes estimator of $\hat{\text{FDR}}(\lambda )$ for the significant region Λ(*λ*) = {(**w**, **z**) | *R*_ODP_(**w**, **z**) ≥ *λ*} can be obtained by plugging-in the maximum likelihood estimate of **η**.

Note that we conducted small simulations for checking correctness of the proposed ODP procedure and these results were provided in the Supplementary Materials available online at http://dx.doi.org/10.1155/2013/568480.

### 3.4. The Posterior Distribution

Under the semiparametric hierarchical mixture model, the posterior distributions of *θ*_*k*_ and *σ*_*k*_^2^  (*k* = 1,2,…, *m*) can be obtained analytically as noted above; this gives a computational advantage for implementing Bayesian inference. The posterior distribution will be used to derive estimates and confidence intervals of *d*_*k*_'s, in addition to the statistical significance measures such as the *q*-value.

Let *γ*_0*k*_ and *γ*_1*k*_ denote indicator variables to which the *k*th gene component belongs (*k* = 1,2,…, *m*). The joint posterior distribution of (*θ*_*k*_, *σ*_*k*_^2^) is expressed as a mixture of differential and non-differential components:

(20) p(θk,σk2 ∣ wk,zk)  =p(θk,σk2 ∣ wk,zk,γ0k=1)Pr(γ0k=1 ∣ wk,zk)   +p(θk,σk2 ∣ wk,zk,γ1k=1)Pr⁡(γ1k=1 ∣ wk,zk),

where *p*(*θ*_*k*_, *σ*_*k*_^2^ | **w**_*k*_, **z**_*k*_, *γ*_0*k*_ = 1) = *δ*(*θ*)*p*(*σ*_*k*_^2^ | **w**_*k*_, **z**_*k*_). The weight Pr(*γ*_0*k*_ = 1 | **w**_*k*_, **z**_*k*_) = Pr(*H*_0*k*_ | **w**_*k*_, **z**_*k*_) is given in Section 3.3, and Pr(*γ*_0*k*_ = 1 | **w**_*k*_, **z**_*k*_) = 1 − Pr(*γ*_1*k*_ = 1 | **w**_*k*_, **z**_*k*_). The posterior distribution of the differential component is expressed as

(21) p(θk=aj,σk2 ∣ wk,zk,γ1k=1)  =Pr(θk=aj ∣ σk2,wk,zk)p(σk2 ∣ wk,zk),

where

(22) $$\begin{array}{c} \text{Pr}\left(\theta_{k}=a_{j}\;|\;\sigma_{k}^{2},w_{k},z_{k}\right)=\frac{p\left(w_{k},z_{k}\;|\;\theta_{k}=a_{j},\sigma_{k}^{2}\right)p_{j}}{\sum_{l=1}^{L}p\left(w_{k},z_{k}\;|\;\theta_{k}=a_{l},\sigma_{k}^{2}\right)p_{l}}, \end{array}$$

(23) p(σk2 ∣ wk,zk)  =p(wk,zk ∣ σk2)p(σk2)∫0∞p(wk,zk ∣ σk2)p(σk2)dσk2  =∑j=1Lp(wk,zk ∣ θk=aj,σk2)p(σk2)pj∑j=1L∫0∞p(wk,zk ∣ θk=aj,σk2)p(σk2)dσk2pj,

(24) ∫0∞p(wk,zk ∣ θk=aj,σk2)p(σk2)dσk2  =Γ(n/2+α)Γ(α)βα(2π)−n/2   ×(∑i=1n0wki2+∑i=1n1(zki2−aj)22+β)−(n/2+α).

The marginal posterior distribution of *θ*_*k*_ can be obtained as follows:

(25) Pr(θk=aj ∣ wk,zk)=p(wk,zk ∣ θk=aj)Pr(θk=aj)∑l=1Lp(wk,zk ∣ θk=al)Pr(θk=al)=((∑i=1n0wki2+∑i=1n1(zki2−aj)2)/2+β)−(n/2+α)pj∑l=1L{((∑i=1n0wki2+∑i=1n1(zki2−al)2)/2+β)−(n/2+α)pl}.

## 4. Applications

We illustrate our method using real data sets from two clinical studies with microarrays. Because previous simulation studies have indicated that the Storey's method [10] is more powerful than many other multiple testing methods [10, 12, 35], we chose to use this method as a reference.

### 4.1. Prostate Cancer Example

**Figure 1:**
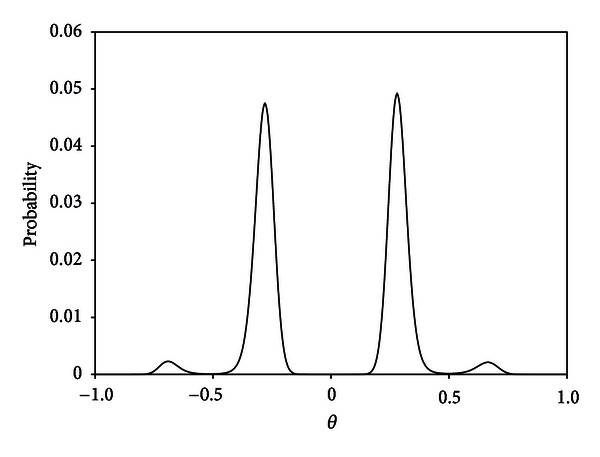
The nonparametric estimate for the differential component of the prior distribution of the prostate cancer data [38].

Efron [36, 37] analyzed a modified gene expression data set from Singh et al. [38] with *m* = 6033 genes for *n* = 102 samples; *n*_1_ = 52 prostate cancer patients and *n*_0_ = 50 healthy controls. For the proposed empirical Bayes approach, we placed the grid points for *G*(*θ*) equally spaced on [−1, 1] by 0.01 (except for 0; *L* = 200). The hyperparameters were estimated as ${\hat{\pi }}_{0}=0.716$,$\;\;{\hat{\pi }}_{1}=0.284$,$\;\;\hat{\alpha }=8.624$,$\;\;\text{and}\;\;\hat{\beta }=7.373$, and the estimated prior distribution for *G*(*θ*) is given in Figure 1. The estimated prior distribution was skewed and multimodal. This indicates that analytically tractable parametric models (for example, the normal distribution) might be inadequate in this case.

**Figure 2:**
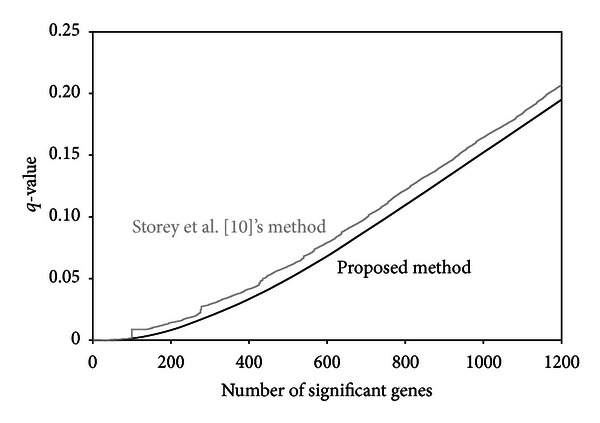
Results of the multiple testing for the prostate cancer data [38].

Figure 2 presents the results of multiple testing by the method of Storey et al. [10] and the empirical Bayes method developed in this article. This plot summarizes the correspondence of the numbers of significant genes and *q*-values [6, 7] for varying values of the cut-off *λ*. For a fair comparison, the same FDR-controlling method presented in Section 3.3 was applied to these two methods. For any levels of the *q*-values, the number of significant genes identified by the proposed method was greater than that of the Storey et al. [10] method.

### 4.2. Lymphoma Example

Dave et al. [39] analyzed Affymetrix HG-U133A and U133B (Affymetrix, Santa Clara, CA) microarray data for 191 biopsy specimens from patients with untreated follicular lymphoma with *m* = 44,928. The data are available from the BRB-ArrayTools Data Archive for Human Cancer Gene Expression [40]. We compared patients who died within 5 years (poor prognosis; *n*_1_ = 51) with patients who survived for more than 5 years (good prognosis; *n*_0_ = 109).

**Figure 3:**
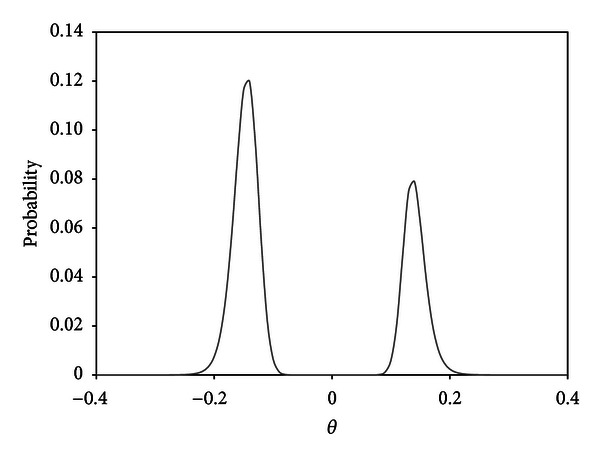
The nonparametric estimate for the differential component of the prior distribution of the lymphoma data [39].

**Figure 4:**
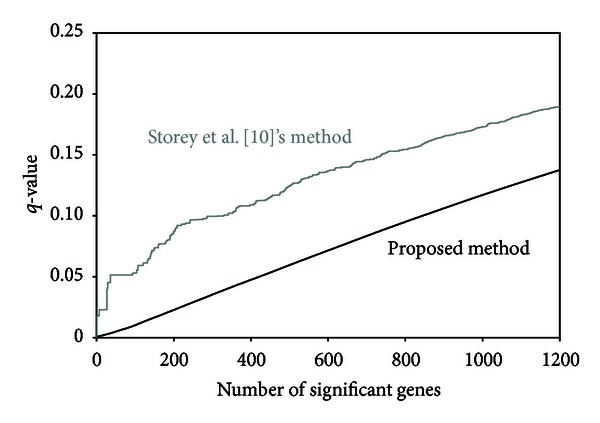
Results of the multiple testing for the lymphoma data [39].

For the maximum likelihood estimate of the semiparametric prior distribution, the hyperparameters were estimated as ${\hat{\pi }}_{0}=0.831,\;\;{\hat{\pi }}_{1}=0.169,\;\;\hat{\alpha }=1.266,\;\;$and$\;\;\hat{\beta }=0.311$. The estimated prior distribution for *G*(*θ*) is given in Figure 3. (The grid points were placed on [−1, 1] equally spaced by 0.01 except for 0; *L* = 200.) Compared with the prostate cancer data, large masses of the estimated *G*(*θ*) are located in the regions of small *θ*, and thus the signal on differential expression contained in the data would be weaker than that in the prostate cancer data. In Figure 4, the relationships of the numbers of significant genes and their *q*-values are presented. The number of significant genes identified by the empirical Bayes method was greater than that of the method of Storey et al. [10].

## 5. Discussion

For efficient gene screening using high-dimensional microarray data, multiple testing methodologies are effective tools for controlling false positives findings. Although the multiple testing can provide a relevant framework to ensure control of false positives for a set of significant genes (e.g., FDR [5–7]), researchers would like to find as many true positive genes as possible for further investigations. The optimal discovery procedure (ODP), introduced by Storey [8], would provide an efficient solution for this requirement. Storey et al. [10] provided desirable results in many numerical evaluations [10, 12, 35].

In this article, we proposed an empirical Bayes ODP method based on a semiparametric hierarchical model, through relaxing the parametric prior assumption in the empirical Bayes ODP method by Noma and Matsui [14]. Hierarchical mixture models and empirical Bayes methods form a basis for information sharing across genes and provide a framework for efficient multiple testing [15–21]. Although empirical Bayes methods are more robust to prior misspecifications than eliciting a single prior, parametric empirical Bayes methods can suffer from a lack of robustness when the true prior is not from the assumed parametric family, as pointed out by Morris [28], such as a failure to incorporate the mixture structure or specify the form of the effect-size distribution (*G*). Actually, the estimated prior distribution in the prostate cancer example (Section 4.1) had a multimodal and skewed shape. In such examples, natural conjugate families might be inadequate for modeling an unknown random effects distribution. The “smoothing-by-roughening” approach [22, 23] is one of the flexible modeling methods in empirical Bayes inference [41–43] and could be a basis for effective empirical Bayes inference for a broad range of situations. An extension of our method is to specify a nonparametric distribution for the random effect distribution of *σ*_*k*_^2^ through applying the smoothing-by-roughening approach, although it requires substantial computation for estimating a large number of parameters and can suffer from unstable parameter estimates.

Previously, many efficient gene selection methods have been proposed for microarray studies, not only the multiple testing methodologies, for example, Bayesian ranking methods [20, 21, 43]. For evaluating practical values of these methods, comprehensive numerical investigations, including large-scale simulations and applications to many real datasets, would be a worthwhile subject.

## Supplementary Material

Small simulation results for evaluating the validity of the proposed method and its performances compared with the Storey et al. (Biostatistics 2007; 8: 347-368)'s method.

## Appendix

### A. The EM Algorithm

The maximum likelihood estimate of the hyperparameters **η** is obtained using the EM algorithm [31]. Regarding *θ*_*k*_s, *σ*_*k*_^2^s, *γ*_0*k*_s, and *γ*_1*k*_s as missing variables, the log complete data likelihood is expressed as

(A.1) log⁡L(η)=∑k=1mγ0klog⁡p(wk,zk,σk2 ∣ η,γ0k=1)  +γ1klog⁡p(wk,zk,θk,σk2 ∣ η,γ1k=1)  +γ0klog⁡π0+γ1klog⁡π1=∑k=1mγ0klog⁡p(wk,zk ∣ σk2,γ0k=1)  +γ1klog⁡p(wk,zk ∣ θk,σk2,γ1k=1)  +γ1k logPr(θk=aj ∣ η,γ1k=1)  +log⁡p(σk2 ∣ η)+γ0klog⁡π0+γ1klog⁡π1.

The first and second terms in the sum do not depend on the hyperparameters. Thus the objective function in each *M*-step is given by

(A.2) Q(η ∣ η(t))=∑k=1mE[γ1klog⁡ Pr(θk=aj ∣ η,γ1k=1)     +log⁡p(σk2 ∣ η)     +γ0klog⁡π0+γ1klog⁡π1 ∣ wk,zk,η(t)]=∑k=1mω1k(t)E[logPr(θk=aj ∣ η,γ1k=1) ∣ wk,zk,η(t)]  +E[log⁡p(σk2 ∣ η) ∣ wk,zk,η(t)]  +ω0k(t)log⁡π0+ω1k(t)log⁡π1,

where *ω*_0*k*_^(*t*)^ = Pr(*γ*_0*k*_ = 1 | **w**_*k*_, **z**_*k*_), *ω*_1*k*_^(*t*)^ = Pr(*γ*_1*k*_ = 1 | **w**_*k*_, **z**_*k*_). The superscripts indicate the number of the iterations. Solving the optimization problem, the *M*-step is expressed as

(A.3) π0(t+1)=1m∑k=1mω0k(t),  π1(t+1)=1−π0(t+1),pj(t+1)=(∑k=1mω1k(t)pj(t)(∑i=1n0wki2+∑i=1n1(zki2−aj)22+β(t))−(n/2+α(t))) ×({∑k=1mω1k(t)}∑l=1L{pl(t)(∑i=1n0wki2+∑i=1n1(zki2−al)22   +β(t))−(n/2+α(t))})−1.

Also, the optimization problem for *α* and *β* is equivalent to the maximum likelihood estimation of the shape and scale parameters for the inverse gamma distribution. It can be solved simply by any numerical optimization methods provided in standard software (e.g., nlm or optim in R).
