# Supplementary material for: An Empirical Bayes Optimal Discovery Procedure Based on Semiparametric Hierarchical Mixture Models
Source: Comput Math Methods Med. 2013 Apr 10;2013:568480. doi: 10.1155/2013/568480 (PMC3649332; doi:10.1155/2013/568480)
Supplement: Supplementary file 1 — Small simulation results for evaluating the validity of the proposed method and its performances compared with the Storey et al. (Biostatistics 2007; 8: 347-368)'s method. [file 568480.f1.pdf]

Supplementary Materials for

An empirical Bayes optimal discovery procedure based on semiparametric hierarchical mixture models

Hisashi Noma* and Shigeyuki Matsui
Department of Data Science, The Institute of Statistical Mathematics
10-3 Midori-cho, Tachikawa, Tokyo 190-8562, Japan
*e-mail: noma@ism.ac.jp

In this supplementary material, we provide small simulation results for checking the correctness of our proposed empirical Bayes method. We assessed the performances by comparing *average power*, which is defined as ETP/m_1 for a certain *average type I error rate*, EFP/m_0, with the Storey (2007) *et al.*'s method. Simulation data were generated using the two-stage sampling model presented in Section 3,

$$x_{k1},\ldots,x_{kn_0} \sim \mathrm{N}(\mu_{0k}, \sigma_k^2)$$
$$y_{k1},\ldots,y_{kn_1} \sim \mathrm{N}(\mu_{1k}, \sigma_k^2)$$

with a prior distribution

$$\mu_{0k} \sim \mathrm{N}(0, 1) \qquad (k = 1,2,\ldots,2000)$$
$$d_k \sim \pi_0\, \delta(\theta) + \pi_1\, \mathrm{N}(0.40, 0.10^2)$$
$$\sigma_k^2 \sim \mathrm{Gamma}^{-1}(1.00, 0.02).$$

We considered equal sample sizes between classes, that is, $n_0 = n_1 = n / 2$, with $n = 20$ or 40. We conducted 100 simulations for each setting. Simulation results are presented at Figure S1. The average power of the poposed method was greater than that of the Storey *et al.* (2007)'s method, uniformly.

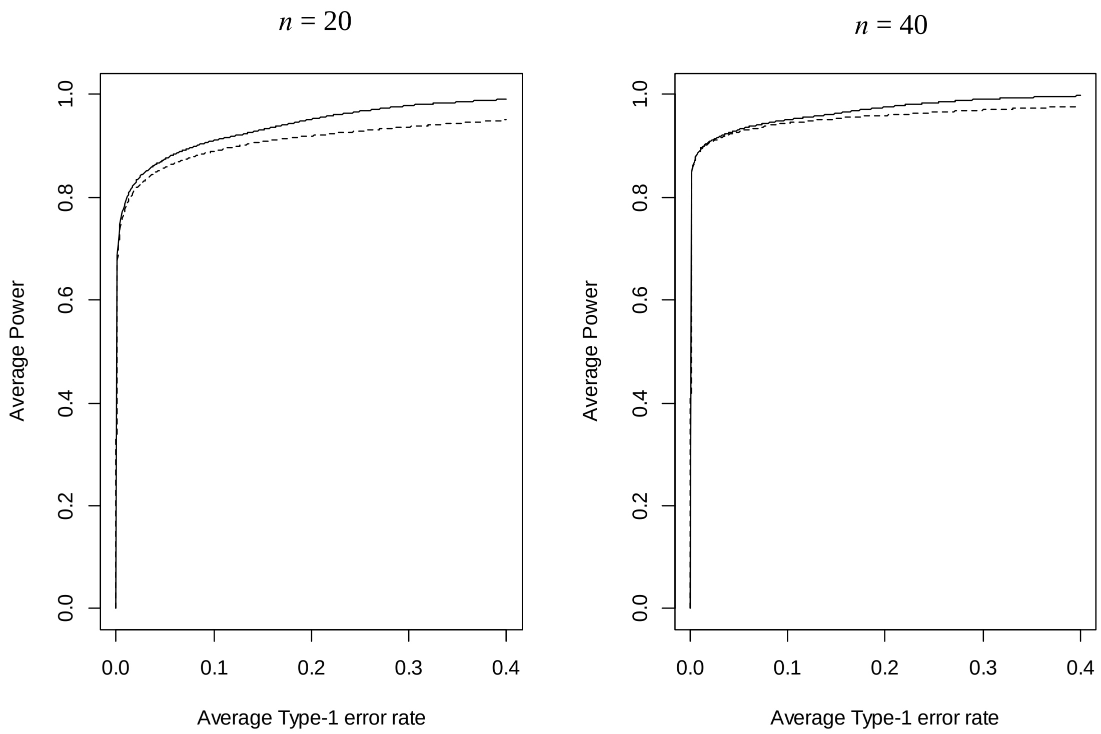

Figure S1. Simulation results: The proposed semiparametric empirical Bayes method (solid line) and the Storey *et al*. (2007)'s method (dashed line).

Reference

Storey, J. D., Dai, J. Y., Leek, J. T. (2007). The optimal discovery procedure for large-scale significance testing, with applications to comparative microarray experiments. *Biostatistics* **8**: 347-368.
